# Supplementary material for: AGMO Inhibitor Reduces 3T3-L1 Adipogenesis
Source: Cells. 2021 May 1;10(5):1081. doi: 10.3390/cells10051081 (PMC8147360; doi:10.3390/cells10051081)
Supplement: Supplementary file 1 [file cells-10-01081-s001.zip › cells-1170195-supplementary.pdf]

Supplementary Methods, Tables and Figures

Supplementary Tables

Suppl. Table S1

Candidate compounds

Chemical name	Structure
**Cp1**<br>**3-[(2-amino-6,7-dimethyl-4-oxo-4,6,7,8-tetrahydro-5(3H)-pteridinyl)carbonyl]-1-octylpyridinium**	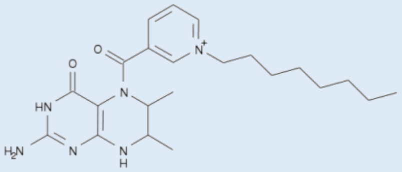
**Cp2**<br>**2-amino-5-(3-pyridinylcarbonyl)-6-(2,2,5-trimethyl-1,3-dioxolan-4-yl)-5,6,7,8-tetrahydro-4(3H)-pteridinone**	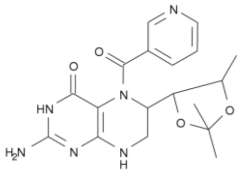
**Cp3**<br>**2-amino-6-(1,2-dihydroxypropyl)-5-(3-pyridinylcarbonyl)-5,6,7,8-tetrahydro-4(3H)-pteridinone**	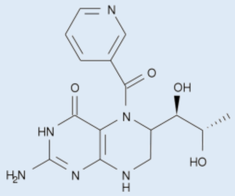
**Cp4**<br>**N-[6,7-dimethyl-4-oxo-5-(3-pyridinylcarbonyl)-3,4,5,6,7,8-hexahydro-2-pteridinyl]-2-methylpropanamide**	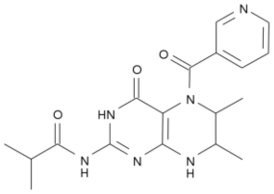
**Cp5**<br>**2-amino-6,7-dimethyl-5-(3-pyridinylcarbonyl)-5,6,7,8-tetrahydro-4(3H)-pteridinone**	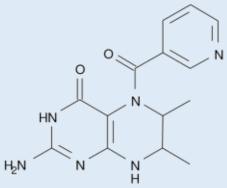
**Cp6**<br>**2-(dimethylamino)-6,7-dimethyl-5,6,7,8-tetrahydro-4(3H)-pteridinone**	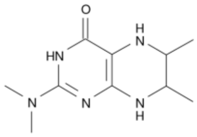
**Cp7**<br>**N-(2,4-diamino-6-methoxy-5-pyrimidinyl)-4-methylbenzenesulfonamide**	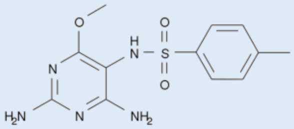
**Cp8**<br>**6-amino-2-(propylsulfanyl)-4-pyrimidinol**	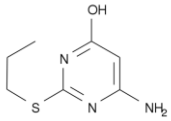

**Cp9**<br>**N-(4,6-dimethoxypyrimidin-2-yl)-N,N-dimethylamine**	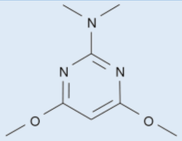
**Cp10**<br>**N-(4,6-dimethoxy-2-pyrimidinyl)-3-(4-methoxyphenyl)propanamide**	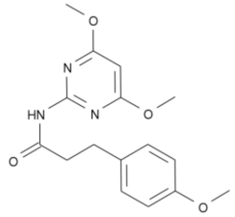
**Cp11**<br>**N-(4,6-dimethoxy-2-pyrimidinyl)-2,2-diphenylacetamide**	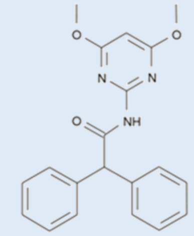
**Cp12**<br>**5-ethoxy-2,4-quinazolinediamine**	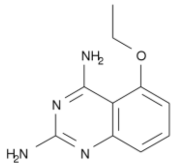
**Cp13**<br>**4-methoxy-6-phenyl-2-pyrimidinylamine**	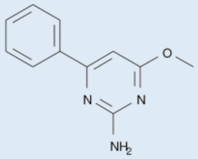
**Cp14**<br>**N-[2-(2-methoxyanilino)-4-pyrimidinyl]-N-(2-methoxyphenyl)amine**	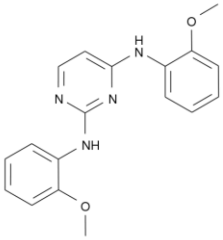
**Cp15**<br>**4-methoxy-6-(4-methoxyphenyl)-2-pyrimidinylamine**	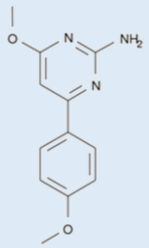
**Cp16**<br>**6-amino-5-[(4-hydroxybenzylidene)amino]-4-pyrimidinol**	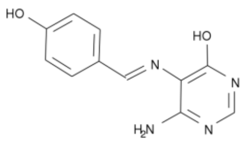

**Cp17**<br>**5,6-diamino-2-phenyl-4-pyrimidinol**	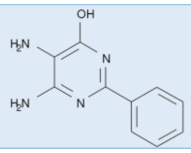
**Cp18**<br>**3-[({6,7-dimethyl-5-[(1-methyl-3-pyridiniumyl)carbonyl]-4-oxo-3,4,5,6,7,8-hexahydro-2-pteridinyl}amino)carbonyl]-1-methylpyridinium**	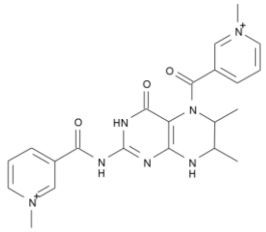

Suppl. Table S2

Stable RAW264.7 cell lines with AGMO-knockdown or AGMO-overexpression

Cell line name	RAW shLUC	RAW shAGMO506	RAW SFFV +huAGMO
**Original cell line**	RAW 264.7 (ATCC® TIB-71™)	RAW 264.7 (ATCC® TIB-71™)	RAW 264.7 (ATCC® TIB-71™)
**Vector name**	pHR-shLUC Puro<br>(Lentiviral Expression Vector)	pHR-shAGMO506 Puro<br>(Lentiviral Expression Vector)	pHR-huAGMO-ires Puro<br>(Lentiviral Expression Vector)
**Vector backbone**	pHR	pHR	pHR
**Inserts**	Short hairpin RNA (shRNA): Targeting 155–173 from pGL3 Luciferase (Promega)<br>Puromycin N-acetyl transferase (pac) gene (Puromycin resistance)	Short hairpin RNA (shRNA) :<br>5’-GAGGTGCCTGATTACGTAA-3’<br>Targeting murine AGMO 506–524 (NM_178767.5)<br>Puromycin N-acetyl transferase (pac) gene (Puromycin resistance)	SFFV promotor (instead of CMV)<br>Human AGMO ORF (NM_001004320.1) with C-terminal 3xFLAG<br>IRES (Internal ribosome entry site)<br>Puromycin N-acetyl transferase (pac) gene (Puromycin resistance)
**Integration into genome**	Inserts are flanked by LTR for stable integration into RAW 264.7 genome	Inserts are flanked by LTR for stable integration into RAW 264.7 genome	Inserts are flanked by LTR for stable integration into RAW 264.7 genome

Stable knockdown RAW264.7 cell lines were created as described in [1] and were kindly provided by Ernst R. Werner and Katrin Watschinger, Institute of Biological Chemistry, Biocenter, Medical University Innsbruck. Control cells express shRNA targeting luciferase (shLUC). The express endogenous AGMO. shLUC was replaced with shAGMO to generate stable AGMO knockdown cells (shAGMO) or transfection with an expression vector for human AGMO tagged with a FLAG-tag (+huAGMO), so that they express the endogenous murine AGMO plus human AGMO.

Suppl. Table S3

3A. QRT-PCR primer sequences

Gene	Species	Sense (5'→3')	Antisense (5'→3')
AGMO (TMEM195)	Mouse	CTTTCTTAGGAGTTGACTTTGGCTACT	TGTGCTGCCCAGAAAATATTAATC
AGMO (TMEM195)	Human	CTGACCTTGACTTCCATTGGATT	CAAGCAACGGAGAGTTTCCATA
Arg1	Mouse	ACGGCAGTGGCTTTAACCTT	AGGTAGTCAGTCCCTGGCTT
CCNA2(CyclinA2)	Mouse	GCCTTCACCATTCATGTGGAT	TTGCTGCGGGTAAAGAGACAG
CCNB1(CyclinB1)	Mouse	CTTGCAGTGAGTGACGTAGAC	CCAGTTGTCGGAGATAAGCATAG
CCND2 (CyclinD2)	Mouse	GAGTGGGAACTGGTAGTGTTG	CGCACAGAGCGATGAAGGT
CCND3 (CyclinD3)	Mouse	CGAGCCTCCTACTTCCAGTG	GGACAGGTAGCGATCCAGGT
CCNE1 (CyclinE1)	Mouse	GTGGCTCCGACCTTTCAGTC	CACAGTCTTGTCAATCTTGGCA
CD206 (Mrc1)	Mouse	CCATCTCAGTTCAGACGGCA	ACGGAAGCCCAGTCAGTTTT
CEBPA (CEBPα)	Mouse	CAAGAACAGCAACGAGTACCG	GTCACTGGTCAACTCCAGCAC
Eef2	Mouse	AGGCCTGTGTAATATAGCTGCG	CTCTGTGTAGTTTGTAGCTCTGTCT
Gch1	Mouse	CCGCTTACTCGTCCATTCTGC	CCTTCACAATCACCATCTCGTCA
Il23a (IL-23a)	Mouse	TGTGCCCCGTATCCAGTGT	CGGATCCTTTGCAAGCAGAA
Il6 (IL-6)	Mouse	CCGGAGAGGAGACTTCACAG	TTCTGCAAGTGCATCATCGT
Nos3 (eNOS)	Mouse	TCAGCCATCACAGTGTTCCC	ATAGCCCGCATAGCGTATCAG
Pp1a	Mouse	GCTGGACCA-AACACAAACGG	GCCATTCCTGGACCCAAAAC
PPARG (PPARγ)	Mouse	TCGCTGATGCACTGCCTATG	GAGAGGTCCACAGAGCTGATT
Tnfa (TNF-α)	Mouse	TGCCTATGTCTCAGCCTCTT	GAGGCCATTTGGGAACTTCT
Wls (Wntless)	Mouse	ATGGCTGGGGCAATTATAGAAAA	GGGTGCTGGAGCGATCAAG
Wnt10b	Mouse	GAAGGGTAGTGGTGAGCAAGA	GGTTACAGCCACCCCATTCC
Wnt5b	Mouse	CTGCTGACTGACGCCAACT	CCTGATACAACTGACACAGCTTT

3B. QRT-PCR commercial primers

Gene	Species	Vendor	Product#
Nos2 (iNOS)	Mouse	QIAGEN	PPM02928B
Tgm2	Mouse	QIAGEN	PPM40872F
Alox15	Mouse	QIAGEN	PPM25132A

Suppl. Table S4

4A. Primary antibodies for Western Blots

Antibody	Host	Species reactivity	Vendor	Product#	Dilution
**β-actin**	Rabbit	Mouse	Sigma	A2066	1:1000
**β-actin**	Mouse	Mouse	Sigma	A5441	1:1000
**AGMO**	Rabbit	Human, Mouse	Proteintech	21355-1-AP	1:200
**FLAG-Tag**	Mouse	Mouse	Sigma	F1804	1:200
**Calnexin**	Mouse	Human, Mouse	Abcam	ab31290	1:200

4B. Primary antibodies for immunofluorescence

Primary antibody	Host	Species reactivity	Vendor	Product#	Dilution
**AGMO**	Rabbit	Human, Mouse	Abcam	ab87236	1:250
**FLAG-tag**	Mouse	Mouse	Sigma	F1804	1:200
**PDI/P4HB**	Mouse	Mouse	Abcam	ab2792	1:100
**Pref-1 (DLK1)**	Rat	Mouse, Rat	R&D	D187-3	1:500
**FABP4**	Goat	Mouse	MBL	AF1443	1:100

4C. Secondary antibodies

Fluorophor	Host	Species reactivity	Vendor	Product#	Dilution
**Alexa Fluor 488**	Goat	Rabbit	Invitrogen	A27034	1:800
**Alexa Fluor 488**	Goat	Chicken	Invitrogen	A11039	1:800
**Alexa Fluor 488**	Goat	Mouse	Invitrogen	A11001	1:800
**Alexa Fluor 488**	Goat	Rat	Invitrogen	A11006	1:800
**Alexa Fluor 546**	Goat	Rat	Invitrogen	A11081	1:800
**Cy3**	Rabbit	Goat	Sigma	C2821	1:800
**Cy3**	Sheep	Mouse	Sigma	C2181	1:800
**Cy3**	Sheep	Rabbit	Sigma	C2306	1:800
**IRDye® 800CW**	Goat	Rabbit	LI-COR	926-32213	1:10 000 (WB)
**IRDye® 800CW**	Goat	Mouse	LI-COR	926-32210	1:10 000 (WB)
**IRDye® 680RD**	Goat	Rabbit	LI-COR	926-68071	1:10 000 (WB)
**IRDye® 680RD**	Goat	Mouse	LI-COR	926-68070	1:10 000 (WB)

Supplementary Figures

Suppl. Fig. S1: Synthesis of AGMO substrate

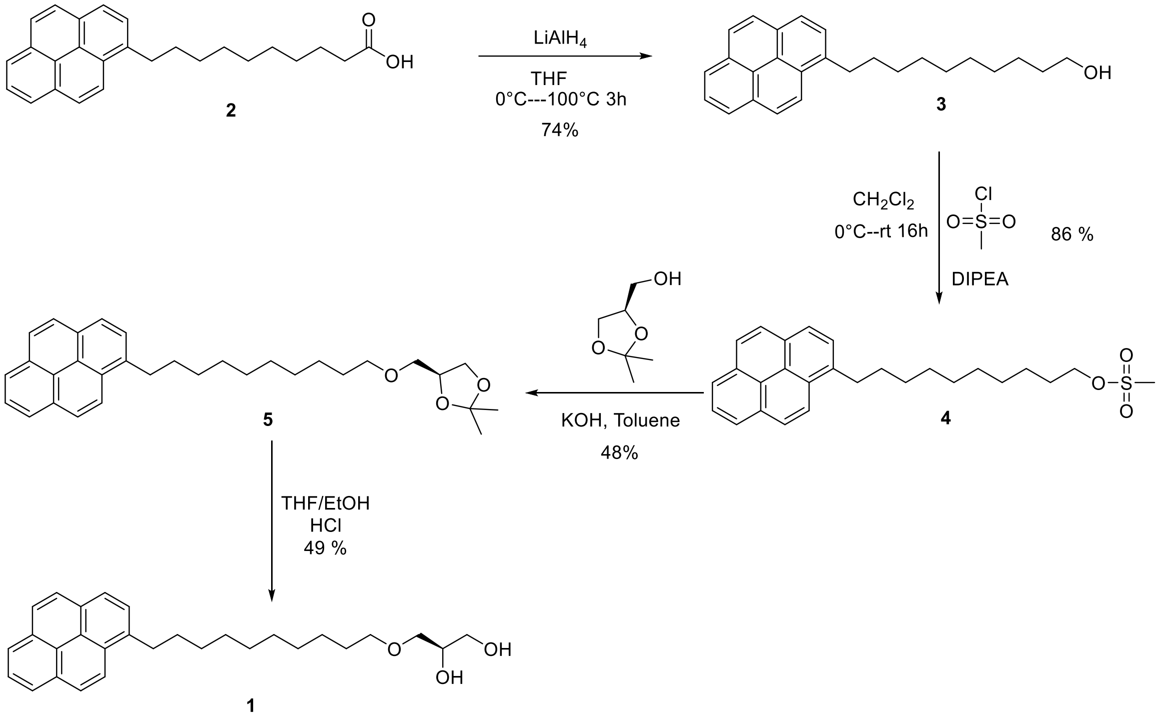

Illustration of the steps and intermediates to generate the AGMO substrate

AGMO substrate (R)-3-((10-(pyren-1-yl)decyl)oxy)propane-1,2-diol (short: 1-Pyrenedecylglycerol) **(1)** was generated in a 4-step process. Individual steps are explained below.

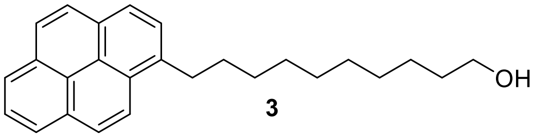

10-(Pyren-1-yl)decan-1-ol (3)

Lithium aluminum hydride (123 mg, 3.24 mmol) was added step-wise to a stirred solution of 10-(pyren-1-yl)decanoic acid (**2**) (200 mg, 0.54 mmol) in dry THF (8 mL) at 0 °C, and the resulting solution was heated to reflux temperature for 3h. The reaction mixture was then cooled to room temperature (rt), and water was carefully added to quench the reaction. The aqueous phase was then extracted with ethyl acetate (3x). The combined organic extracts were washed with brine, dried over magnesium sulfate, filtered and evaporated under reduced pressure. The residue was purified by flash chromatography (hexane/ethyl acetate, 9:1) to obtain 143 mg (74 %) of compound **3**. ^{1}H NMR (400 MHz, DMSO-d_6) δ 8.33 (d, *J* = 9.3 Hz, 1H), 8.26 (dd, *J* = 7.6, 3.3 Hz, 2H), 8.22-8.19 (m, 2H), 8.13 (d, *J* = 9.0 Hz, 1H), 8.10 (d, *J* = 9.0 Hz, 1H), 8.05

(t, J = 7.6 Hz, 1H), 7.93 (d, J = 7.8 Hz, 1H), 4.29 (t, J = 5.2 Hz, 1H), 3.38-3.29 (m, 4H), 1.76 (m, J = 7.4 Hz, 2H), 1.45-1.30 (m, 6H), 1.26-1.21 (m, 8H); LC-MS (m/z): 359 $[M+H]^{+}$.

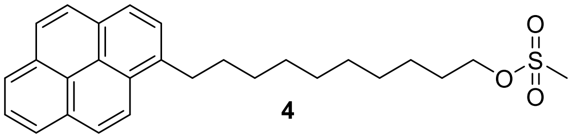

4

10-(Pyren-1-yl)decyl methanesulfonate (4)

Mesyl chloride (60.4 µL, 0.78 mmol) and DIPEA (340 µL, 1.95 mmol) were added to a stirred solution of 3 (140 mg, 0.39 mmol) in dry dichloromethane (8 mL) at 0 °C, and the resulting solution was allowed to stir at rt overnight. The reaction was quenched with a saturated sodium bicarbonate solution and the aqueous phase extracted with dichloromethane (3x). The combined organic extracts were washed with brine, dried over magnesium sulfate, filtered and evaporated under reduced pressure to give 148 mg (86 %) of crude compound 4 that was used in the next step without further purification. 1H NMR (400 MHz, DMSO-d6) δ 8.33 (d, J = 9.4 Hz, 1H), 8.26 (dd, J = 7.6, 3.8 Hz, 2H), 8.22-8.19 (m, 2H), 8.13 (d, J = 9.0 Hz, 1H), 8.10 (d, J = 9.0 Hz, 1H), 8.05 (t, J = 7.6 Hz, 1H), 7.94 (d, J = 7.8 Hz, 1H), 4.15 (t, J = 6.2 Hz, 2H), 3.34-3.29 (m, 2H), 3.14 (s, 3H), 1.77 (m, J = 7.4 Hz, 2H), 1.61 (m, J = 7.0 Hz, 2H), 1.42 (m, J = 7.4 Hz, 2H), 1.35-1.21 (m, 10H); LC-MS (m/z): 437 [M+H]+, 460 [M+Na]+.

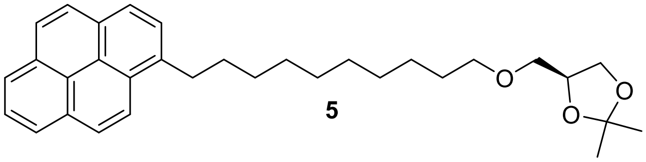

5

(*S*)-2,2-dimethyl-4-(((10-(pyren-1-yl)decyl)oxy)methyl)-1,3-dioxolane (5)

Potassium hydroxide (19.7 mg, 0.35 mmol) was added to a stirred solution of 4 (140 mg, 0.32 mmol) and (S)-(2,2-dimethyl-1,3-dioxolan-4-yl)methanol (44 µL, 0.35 mmol) in toluene (10 mL), and the resulting mixture was heated at reflux temperature for 3.5h. Subsequently, the solvent was evaporated and the residue re-dissolved in water and extracted with ethyl acetate (3x). The combined organic extracts were washed with brine, dried over magnesium sulfate, filtered and evaporated under reduced pressure. The residue was purified by flash chromatography (hexane/ethyl acetate, 9:1) to obtain 73 mg (48 %) of compound 5. 1H NMR (250 MHz, CDCl3) δ 8.27 (d, J = 9.3 Hz, 1H), 8.16-8.06 (m, 4H), 8.16-8.06 (m, 4H), 8.02 (d, J = 9.0 Hz, 1H), 7.98 (d, J = 9.0 Hz, 1H), 7.96 (t, J = 7.5 Hz, 1H), 7.85 (d, J = 7.8 Hz, 1H), 4.25 (m, J = 6.2 Hz, 1H), 4.04 (dd, J = 8.2, 6.4 Hz, 1H), 3.71 (dd, J = 8.2, 6.4 Hz, 1H), 3.52-3.38 (m, 4H), 3.35-3.28 (m, 2H), 1.84 (m, J = 7.6 Hz, 2H), 1.58-1.42 (m, 2H), 1.41 (s, 3H), 1.35 (s, 3H), 1.41-1.24 (m, 12H); LC-MS (m/z): 473 [M+H]+.

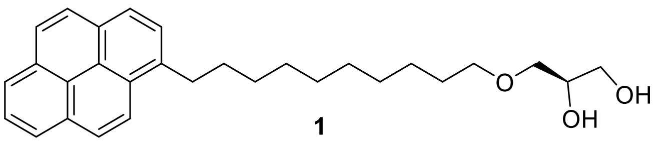
1

(*R*)-3-((10-(pyren-1-yl)decyl)oxy)propane-1,2-diol (1)

1 mL of hydrochloric acid 1M solution was added to a stirred solution of 5 (71 mg, 0.15 mmol) in THF/ethanol (1.5 mL, 2:1), and the resulting mixture was stirred at rt overnight. The reaction was quenched with a saturated sodium bicarbonate solution and the aqueous phase extracted with ethyl acetate (3x). The combined organic extracts were washed with brine, dried over magnesium sulfate, filtered and evaporated under reduced pressure. The residue was purified by preparative reversed phase HPLC (C-18, acetonitrile/water with 0.1 % formic acid) to obtain 32 mg (49 %) compound 1. 1H NMR (300 MHz, CDCl3) δ 8.28 (d, J = 9.3 Hz, 1H), 8.16-8.06 (m, 4H), 8.17-8.08 (m, 4H), 8.04 (d, J = 9.0 Hz, 1H), 7.99 (d, J = 9.0 Hz, 1H), 7.98 (t, J = 7.5 Hz, 1H), 7.87 (d, J = 7.8 Hz, 1H), 3.88-3.82 (m, 1H), 3.72 (dd, J = 11.4, 3.8 Hz, 1H), 3.64 (dd, J = 11.4, 5.1 Hz, 1H), 3.55-3.41 (m, 4H), 3.36-3.31 (m, 2H), 2.34 (br s, 2H), 1.85 (m, J = 7.6 Hz, 2H), 1.59-1.43 (m, 4H), 1.39-1.26 (m, 12H); 13C NMR (300 MHz, CDCl3) δ 137.3, 131.4, 130.9, 129.6, 128.6, 127.5, 127.2, 127.0, 126.4, 125.7, 125.0, 124.7, 124.6, 123.5, 72.5, 71.8, 70.3, 64.3, 33.6, 31.9, 29.8, 29.5(5x), 29.4, 26.0; Rf HPLC: 11.61 Min (13 Min form 10 to 95% MeCN in water with 0.1 % formic acid) >99.0 % purity; HRMS MALDI-TOF (m/z) 432.2654 [M+H]+ (calc. C29H37O3 432.2664).

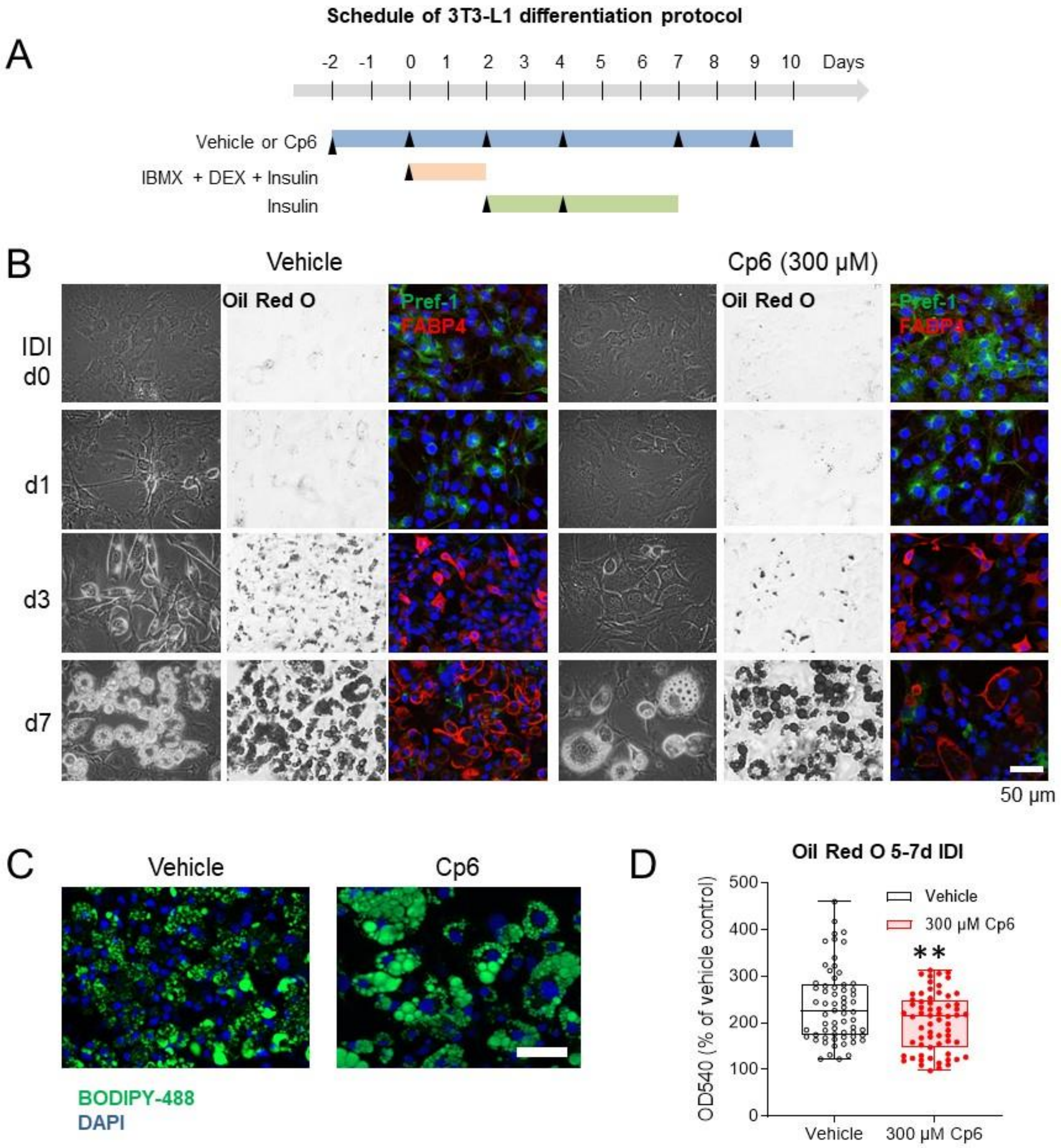

Suppl. Fig. S2.

Effects of Cp6 at 300 µM on adipocyte differentiation

(**A**): Flowchart showing the differentiation procedure. Cells were treated with vehicle (0.1% DMSO) or 300 µM Cp6. The differentiation medium (IDI+) consisted in dexamethasone (DEX), 3-isobutyl-1-methylxanthine (IBMX) and insulin. (**B**): Time course of the morphology and expression of pre/adipocyte markers in 3T3-L1 pre/adipocytes. FABP4 (fatty acid binding protein) is a marker for mature adipocytes, and Pref-1 (preadipocyte factor) is a marker for preadipocytes. (**C**) Immunofluorescent staining of lipid droplets with BODIPY-488 at day 7 of the differentiation (+IDI). (**D**) Quantification of lipid droplets per Oil-Red staining at days 5 and 7 of +IDI (pooled time points). Groups were compared with unpaired, 2-sided t-test, **P<0.01.

Suppl. Fig. S3

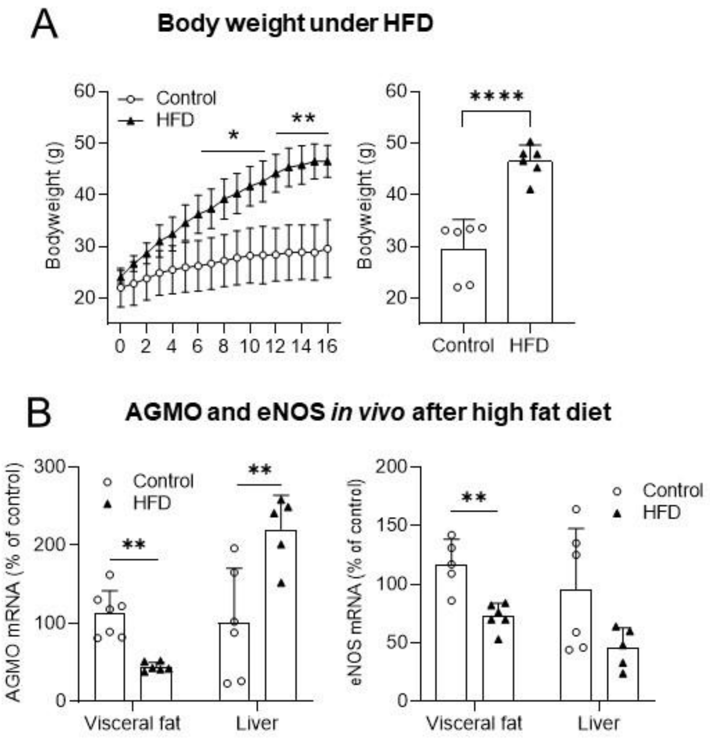

Body weight and AGMO expression in liver and fat under high fat diet (HFD)

A: Time course and final body weights of mice fed with a high fat diet.

B: QRT-PCR analysis of AGMO and eNOS mRNA expression in visceral fat tissue und liver in mice exposed to 16 weeks of a high-fat diet (HFD) versus a standard diet. Eukaryotic elongation factor 2 (Eef2) was used as housekeeping gene, and AGMO/eNOS mRNA was normalized to standard-fed mice set to 100% (for each tissue separately). Bar/scatter show means ± SD and results of individual mice (n = 5-12). Groups were compared per independent, 2-tailed t-test for each tissue separately; **p=0.0056, ****p<0.0001.

Reference

1. Watschinger, K.; Keller, M.A.; McNeill, E.; Alam, M.T.; Lai, S.; Sailer, S.; Rauch, V.; Patel, J.; Hermetter, A.; Golderer, G.*, et al.* Tetrahydrobiopterin and alkylglycerol monooxygenase substantially alter the murine macrophage lipidome. *Proc. Natl. Acad. Sci. U. S. A.* **2015**, *112*, 2431-2436.
